# C-Reactive Protein (CRP), Interferon Gamma-Inducible Protein 10 (IP-10), and Lipopolysaccharide (LPS) Are Associated with Risk of Tuberculosis after Initiation of Antiretroviral Therapy in Resource-Limited Settings

**DOI:** 10.1371/journal.pone.0117424

**Published:** 2015-02-26

**Authors:** Mark W. Tenforde, Nikhil Gupte, David W. Dowdy, David M. Asmuth, Ashwin Balagopal, Richard B. Pollard, Patcharaphan Sugandhavesa, Javier R. Lama, Sandy Pillay, Sandra W. Cardoso, Jyoti Pawar, Breno Santos, Cynthia Riviere, Noluthando Mwelase, Cecilia Kanyama, Johnstone Kumwenda, James G. Hakim, Nagalingeswaran Kumarasamy, Robert Bollinger, Richard D. Semba, Thomas B. Campbell, Amita Gupta, for the ACTG PEARLS and NWCS 319 Study Group

**Affiliations:** 1 Johns Hopkins University School of Medicine, Baltimore, Maryland, United States of America; 2 Johns Hopkins University Bloomberg School of Public Health, Baltimore, Maryland, United States of America; 3 University of California Davis Medical Center, Sacramento, California, United States of America; 4 Research Institute for Health Sciences, Chiang Mai University, Chiang Mai, Thailand; 5 Asociación Civil Impacta Salud y Educación (IMPACTA) Peru Clinical Trials Unit, Lima, Peru; 6 Nelson Mandela School of Medicine, University of KwaZulu-Natal, Durban, South Africa; 7 Instituto de Pesquisa Clínica Evandro Chagas, Fundação Oswaldo Cruz, Rio de Janeiro, Brazil; 8 National AIDS Research Institute, Pune, India; 9 Hospital Nossa Senhora da Conceição, Porto Alegre, Brazil; 10 Les Centres Groupe Haïtien d’Etude du Sarcome de Kaposi et des Infections Opportunites (GHESKIO), Port-Au-Prince, Haiti; 11 University of Witwatersrand, Johannesburg, South Africa; 12 University of North Carolina Project, Kamuzu Central Hospital, Lilongwe, Malawi; 13 Malawi College of Medicine-Johns Hopkins University Research Project, Blantyre, Malawi; 14 University of Zimbabwe, Harare, Zimbabwe; 15 Y.R. Gaitonde Centre for AIDS Research and Education (YRG CARE), Chennai, India; 16 University of Colorado Denver School of Medicine, Aurora, Colorado, United States of America; Tulane University, UNITED STATES

## Abstract

**Objective:**

The association between pre-antiretroviral (ART) inflammation and immune activation and risk for incident tuberculosis (TB) after ART initiation among adults is uncertain.

**Design:**

Nested case-control study (n = 332) within ACTG PEARLS trial of three ART regimens among 1571 HIV-infected, treatment-naïve adults in 9 countries. We compared cases (participants with incident TB diagnosed by 96 weeks) to a random sample of controls (participants who did not develop TB, stratified by country and treatment arm).

**Methods:**

We measured pre-ART C-reactive protein (CRP), EndoCab IgM, ferritin, interferon gamma (IFN-γ), interleukin 6 (IL-6), interferon gamma-inducible protein 10 (IP-10), lipopolysaccharide (LPS), soluble CD14 (sCD14), tumor necrosis factor alpha (TNF-α), and CD4/DR+/38+ and CD8/DR+/38+ T cells. Markers were defined according to established cutoff definitions when available, 75th percentile of measured values when not, and detectable versus undetectable for LPS. Using logistic regression, we measured associations between biomarkers and incident TB, adjusting for age, sex, study site, treatment arm, baseline CD4 and log10 viral load. We assessed the discriminatory value of biomarkers using receiver operating characteristic (ROC) analysis.

**Results:**

Seventy-seven persons (4.9%) developed incident TB during follow-up. Elevated baseline CRP (aOR 3.25, 95% CI: 1.55–6.81) and IP-10 (aOR 1.89, 95% CI: 1.05–3.39), detectable plasma LPS (aOR 2.39, 95% CI: 1.13–5.06), and the established TB risk factors anemia and hypoalbuminemia were independently associated with incident TB. In ROC analysis, CRP, albumin, and LPS improved discrimination only modestly for TB risk when added to baseline routine patient characteristics including CD4 count, body mass index, and prior TB.

**Conclusion:**

Incident TB occurs commonly after ART initiation. Although associated with higher post-ART TB risk, baseline CRP, IP-10, and LPS add limited value to routine patient characteristics in discriminating who develops active TB. Besides determining ideal cutoffs for these biomarkers, additional biomarkers should be sought that predict TB disease in ART initiators.

## Introduction

Tuberculosis (TB) is the leading cause of morbidity and mortality after starting antiretroviral therapy (ART) in most low- and middle-income countries, accounting for approximately 50% of deaths in the first year of ART. [1] Several factors have been associated with incident TB post-ART initiation across studies from diverse settings. These include anemia, hypoalbuminemia, low body mass index (BMI), low baseline CD4+ T cell count, and clinical disease stage. [1–10] As these markers are incompletely predictive of TB, there is an urgent need to identify other biomarkers that that can be rapidly measured and that might better correlate with the immunopathologic basis by which TB develops after ART initiation.[1]

In recent years, several novel soluble markers were identified as having important roles in TB immunopathogenesis and association with accelerated HIV disease progression. These include cytokines (interferon-γ [IFN-γ], interleukin-6 [IL-6], tumor necrosis factor—α [TNF-α]) with known importance in TB defense[11,12]; the chemokine interferon-gamma inducible protein-10 (IP-10), which correlates with active TB[13–23]; and markers of impaired gut mucosal integrity and microbial translocation (EndoCAb IgM, lipopolysaccharide [LPS], sCD14), which are associated with HIV disease progression and immune response to ART. [24–27] In addition, markers of T cell activation (HLA-DR+/CD38+ on CD4+ and CD8+ T lymphocytes) are associated with accelerated HIV disease progression[28]and acute phase reactants (C-reactive protein [CRP] and ferritin) with both active TB and HIV disease progression. [29–36] Although these markers have clear immunological roles, the degree to which they can help to further identify individuals at high risk for TB beyond known risk factors in populations of ART initiators remains uncertain. Discovery of relevant soluble biomarkers associated with high risk of TB may improve clinical care by identifying individuals starting ART who need closer follow-up as well as help us to better understand the immunopathologic basis by which TB develops after ART initiation.

To ascertain whether novel soluble biomarkers can help predict TB risk beyond existing, widely available measures, such as BMI and CD4+ T cell count, we conducted a nested case-control analysis from a large multicenter trial of individuals starting ART in nine, mostly TB high-burden, countries.

## Methods

### Participants and Study Design

Our study population was sampled from the Prospective Evaluation of Antiretrovirals in Resource Limited Settings (PEARLS) trial (Adult AIDS Clinical Trials Group [ACTG] A5175, clinicaltrials.gov NCT00084136), an open-label, randomized clinical trial evaluating the non-inferiority of once-daily versus twice-daily dosing of three ART regimens (efavirenz 600 mg daily, lamivudine-zidovudine 150 mg / 300 mg twice daily; atazanavir 400 mg daily, didanosine-EC 400 mg daily, emtricitabine 200 mg daily; efavirenz 600 mg daily, emtricitabine-tenofovir 200 mg / 300 mg daily).[37]

In brief, from May 2005 to July 2007, 1571 treatment-naïve (≤7 days) HIV-1-infected adults with CD4 count < 300 cells/mm^3^ from 9 countries (Brazil [n = 231], Haiti [n = 100], India [n = 255], Malawi [n = 221], Peru [n = 134], South Africa [n = 210], Thailand [n = 100], United States [n = 210], and Zimbabwe [n = 110]) were randomized to one of three ART arms. Exclusion criteria included therapy for acute medical illness, pregnancy, Karnofsky score < 70, conditions thought likely to compromise study participation, and selected laboratory abnormalities including severe anemia (hemoglobin < 7.5 g/dL), AST or ALT >5-fold the upper limit of normal, calculated creatinine clearance <60 mL/min, and absolute neutrophil count <750/μL. A more detailed description of the study can be found elsewhere. [37]

Using a nested case-control design, we identified all participants within the parent trial who were diagnosed with incident pulmonary or extrapulmonary TB by week 96 after ART initiation (cases). Controls were pre-selected as a random sub-sampling of 30 patients (stratified by treatment arm) from each of the 9 countries not diagnosed with pulmonary or extrapulmonary TB by week 96. Fifteen out of these 270 patients were diagnosed with TB during follow-up and became part of the case group (leaving 255 patients in the control group). Those with diagnosed prevalent TB at study entry were excluded from the analysis.

### Ethics Statement

The study followed human experimentation guidelines of the United States Department of Health and Human Services. Written informed consent was obtained from all participants. Institutional review board and ethics committee approval was obtained at Johns Hopkins University as well as all other sites that enrolled patients, including Barranco CRS (Peru), Beth Israel Medical Center AIDS Clinical Trials Unit [ACTU] (USA), Blantyre CRS (Malawi), Chapel Hill CRS (USA), Chennai Antiviral Research and Treatment CRS (India), Chiang Mai University AIDS Clinical Trials Group [ACTG] CRS (Thailand), Cincinnati CRS (USA), Columbia Physician and Surgeons CRS (USA), Cook County Hospital CORE Center (USA), Duke University Medical Center Adult CRS (USA), Durban International CRS (South Africa), Harbor-UCLA CRS (USA), Hospital Nossa Senhora da Conceicao CRS (Brazil), Instituto de Pesquisa Clinica Evandro Chagas CRS (Brazil), Les Centres GHESKIO Clinical Research Site CRS (Haiti), Malawi CRS (Malawi), NARI Clinic at Gadikhana Drive Kotnis Municipal Dispensary CRS (India), NARI Clinic at NIV CRS (India), NARI Pune CRS (India), Northwestern University CRS (USA), New York University HIV/AIDS CRS (USA), Ohio State University CRS (USA), Parirenyatwa CRS (Zimbabwe), Penn Therapeutics CRS (USA), Rush University CRS (USA), San Miguel CRS (Peru), The Mariam Hospital Clinical Research Site CRS (USA), Trillium Health ACTG CRS (USA), UCLA CARE Center CRS (USA), University of California Davis Medical Center ACTU (USA), University of Hawaii at Manoa Leahi Hospital (USA), University of Rochester ACTG CRS (USA), University of Texas Medical Branch ACTU (USA), University of Texas Southwestern Medical Center Amelia Court Continuity Clinic (USA), University of Colorado Hospital CRS (USA), University of Minnesota ACTU (USA), University of Southern California CRS (USA), Vanderbilt Therapeutics CRS (USA), Washington University Therapeutics CRS (USA), Weill Cornell Chelsea CRS (USA), and Wits Helen Joseph Hospital CRS (South Africa).

### Clinical and Laboratory Assessment

A complete blood count, comprehensive metabolic panel, and HIV RNA viral load were obtained no more than 14 days prior to ART initiation, along with a comprehensive history and physical examination. CD4+ T cells count was obtained 7 to 45 days prior to initiation of ART. HIV RNA levels were measured by realtime PCR by the Amplicor HIV-1 Monitor Test, v1.5 (Roche, Branchburg, NJ, USA). Both CD4+ T cell count and HIV RNA testing were performed at laboratories that participated in external quality assurance programs. Detailed clinical history and physical examination were performed at scheduled visits on week 2, 4, 8, then every 4 weeks through week 24, then every 8 weeks through week 96.

Cases were diagnosed across study sites using standardized ACTG definitions. Supporting clinical and laboratory data for each diagnosis was reviewed by a panel of five physician team members, who were blinded to participant identity, clinic site, demographic characteristics, and study treatment. Diagnoses that did not meet ACTG definitions were revised according to the supporting data. Pulmonary TB cases were considered confirmed if *Mycobacterium tuberculosis* (MTB) was cultured from sputum, bronchoalveolar lavage (BAL), or lung tissue. We also included cases of probable pulmonary TB, defined as individuals without a positive culture for MTB but who met all of the following criteria: 1) One or more of the following: Fever >38°C, night sweats, productive cough, hemoptysis, weight loss; 2) Acid-fast bacilli (AFB) identified from sputum, gastric aspirate, BAL fluid, or lung tissue; 3) Abnormal chest x-ray consistent with pulmonary TB; and 4) Multi-drug therapy started for active TB. Extra-pulmonary TB cases, including miliary TB, were considered confirmed if positive culture was obtained for MTB from an extrapulmonary site. We also included cases of probable extrapulmonary TB, defined as the presence of all of the following criteria: 1) One or more of the following: Fever >38°C, night sweats, malaise, weight loss, adenopathy; 2) Positive AFB smear or histopathology from an extrapulmonary site; and 3) Multi-drug therapy initiated for active TB. Cases of “unmasked” TB immune reconstitution inflammatory syndrome (IRIS) were also included and defined using standardized ACTG definitions adopted from Meintjes et al.[38]

### Biomarker Selection and Measurement

We selected soluble markers with established roles in TB immunopathogenesis and/or association with accelerated HIV disease progression. Marker concentrations were measured using stored plasma or serum collected within 14 days of ART initiation. Single laboratories performed testing in batches for each marker to mitigate variability in methodology. At the University of California, Davis, soluble inflammatory cytokines (IFN-γ, IL-6, TNF-α) were measured in plasma using a Luminex multiplex cytokine platform (R&D Systems, Inc, Minneapolis, MN), IP-10 was measured in plasma using commercially available multiplex ELISA-based assays (Meso Scale Discovery, Rockville, MD), and CD4+ and CD8+ T cell activation was measured on available viable peripheral blood mononuclear cells (PBMCs) by quantifying proportions of HLA-DR+/CD38+ cells via flow cytometry. Thailand and India sites did not archive PBMCs for testing. At Johns Hopkins University, CRP was measured using ELISA (CRP Quantikine ELISA, R&D Systems, Minneapolis, MN), ferritin was measured by immunoturbidimetry using Roche kits on a Hitachi 912 clinical analyzer (Roche, Branchburg, NJ, USA), and microbial translocation markers were measured using commercially available ELISA kits for EndoCAb IgM (Cell Sciences, Canton, MA, USA) and sCD14 (R&D Systems, Minneapolis, MN, USA). LPS was measured from plasma samples using a *Limulus* Amebocyte Lysate assay (LONZA, Walkersville, MD, USA) with previously described modifications. [39]

### Statistical Analysis

Baseline characteristics of cases and controls were compared using the non-parametric Mann-Whitney test for continuous variables or Fisher’s exact test for discrete variables. A Kaplan-Meier analysis assessing time to incident TB for cases over the 96 weeks post-ART initiation was performed. Univariate and multivariable logistic regression analyses were used to ascertain associations between baseline pre-ART factors and incident TB post-ART initiation. Multivariable logistic regression models were also fitted to assess the independent association of prior TB, BMI, hemoglobin, albumin and inflammatory and immune activation biomarkers with incident TB. Each multivariable model adjusted for age, sex, treatment arm, study site, baseline CD4 count, and baseline log_10_ viral load. Race was collinear with study site (as assessed by variance inflation factors) and was not included.

BMI was categorized into clinically meaningful categories defined: <18.5 (low), 18.5–25 (normal), and >25 (high). Anemia was defined using World Health Organization (WHO) sex-specific definitions (hemoglobin <12 g/dL in females, <13 g/dL in males) and hypoalbuminemia using standard definition of albumin <3.5 g/dL. Elevated ferritin was defined as ferritin >150 ng/mL for women or >200 ng/mL for men, which has been suggested as a meaningful cutoff by the WHO. [40] Prior history of TB, obtained by self-report, was categorized as yes or no. Plasma LPS was undetectable for the majority of study participants and we defined the cutoff as detectable versus undetectable. The remaining measured markers were available as continuous data and were analyzed as highest quartile (Q4) compared to other quartiles, as no validated thresholds were available to identify meaningful cutoffs in this international HIV-infected population. For CRP, the upper quartile cutoff was 11.72 mg/dL, which we rounded to 12 mg/dL as a more clinically useful cutoff.

To determine the use of baseline characteristics and pre-ART biomarkers in predicting incident TB, a receiver operating curve (ROC) analysis was performed. A base model was constructed with age, gender, study site, prior history of TB, BMI and CD4 counts. Area under the ROC curve (AUC) and corresponding 95% asymptotic normal confidence interval was calculated. We then compared the AUC for this base model with that of additional models in which individual biomarkers and combinations of biomarkers were added to that set of covariates to assess for improvements in AUC. We chose to add biomarkers to the base model that were independently associated (or showed a trend toward association) with incident TB in univariate +/- multivariable models. Data were analyzed using S-plus and STATA statistical software (version 12.0).

## Results

### Characteristics of cases and controls

**Fig 1:**
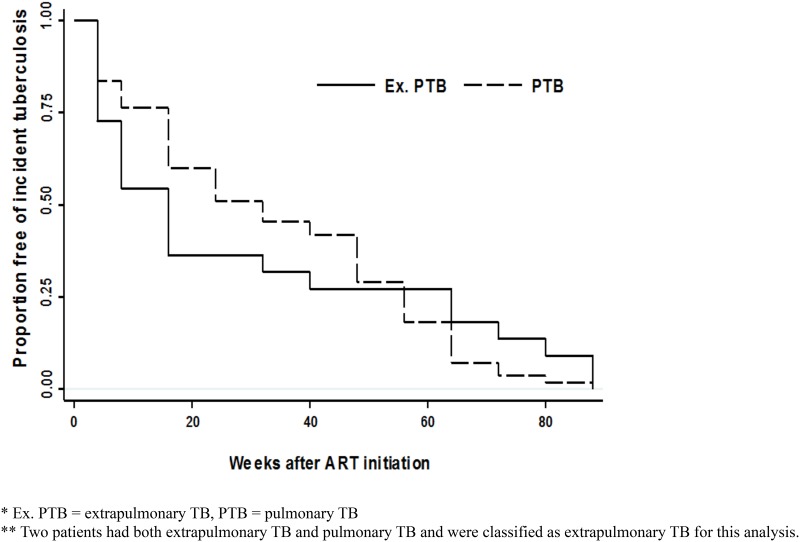
Kaplan-Meier curve of time to TB for persons diagnosed with incident TB by 96 weeks post-ART initiation.

By 96 weeks post-ART initiation, 77 participants were diagnosed with incident TB disease, including 55 with pulmonary and 22 with extrapulmonary TB. Nine cases were associated with IRIS; all were extrapulmonary TB IRIS cases. Median time to diagnosis was 24 weeks. Fig. 1 shows time to TB diagnosis among cases. By country, 4/231 (1.7%) of those from Brazil, 4/100 (4%) from Haiti, 22/255 (8.6%) from India, 14/221 (6.3%) from Malawi, 1/134 (0.7%) from Peru, 21/210 (10.0%) from South Africa, 3/100 (3%) from Thailand, 2/210 (1.0%) from the USA, and 6/110 (5.4%) from Zimbabwe were diagnosed with TB.

Comparisons in baseline characteristics between case and control groups are detailed in Table 1 and Table 2. Additionally, cases had higher baseline median CRP (10.0 mg/dL vs. 3.0 mg/dL, p <0.01) and IP-10 values (2080 pg/mL vs. 1200 pg/mL, p = 0.02) than controls and a trend toward higher median IL-6 values (30.3 pg/mL vs. 23.6 pg/mL, p = 0.09). Median values of other soluble cytokines (IFN-γ and TNF-α), markers of microbial translocation (EndoCAb IgM, LPS, sCD14) and activated CD4+ or CD8+ T cells (HLA+/ DR+/38+) did not differ significantly between groups. In a sub-analysis, we determined the association between baseline factors and TB diagnosed ≤ 24 weeks or >24 weeks post-ART. Baseline median CRP was higher for cases diagnosed in both time periods compared to controls, whereas baseline median IP-10 was only higher in cases diagnosed at ≤ 24 weeks compared to controls.

**Table 1:** Baseline characteristics of cases and controls.

Characteristic	Incident TB	No Incident TB	p-value^1^
	n = 77	n = 255	
Age, years, median (IQR)	34 (29–39)	35 (29–41)	> 0.95
Female at birth, n (%)	31 (40)	126 (49)	0.16
Baseline CD4+ T cell count, cells/mm^3^, median (IQR)	136 (60–201)	180 (90–231)	0.07
Baseline HIV viral load, log_10_copies/mL, median (IQR)	5.2 (4.6–5.7)	5.0 (4.6–5.5)	0.23
Body mass index, kg/m^2^, n (%)			< 0.01
< 18.5	11 (14)	22 (9)	
≥ 18.5–25	58 (75)	165 (65)	
≥ 25	8 (10)	68 (27)	
HIV treatment regimen^2^, n (%)			0.35
EFV+3TC/ZDV	22 (29)	94 (37)	
ATV+DDI+FTC	31 (40)	83 (33)	
EFV+FTC/TDF	24 (31)	78 (31)	
History of TB, n (%)	25 (32)	43 (17)	< 0.01

^1^ Baseline characteristics of cases and controls were compared using the non-parametric Mann-Whitney test for continuous variables or Fisher’s exact test for discrete variables.

^2^ EFV+3TC/ZDV = efavirenz 600 mg daily, lamivudine-zidovudine 150 mg / 300 mg twice daily

ATV+DDI+FTC = atazanavir 400 mg daily, didanosine-EC 400 mg daily, emtricitabine 200 mg daily

EFV+FTC/TDF = efavirenz 600 mg daily, emtricitabine-tenofovir 200 mg / 300 mg daily

**Table 2:** Unadjusted and adjusted odds ratios for the association between selected markers and incident TB within 96 weeks after ART initiation.

Characteristic	Overall	TB Case	TB Control	Unadjusted OR	Adjusted OR^1^
				(95% CI)	(95% CI)
Prior history of TB					
No	264 (80%)	52 (68%)	212 (83%)	1	1
Yes	68 (20%)	25 (32%)	43 (17%)	2.37 (1.33–4.23)	1.09 (0.53–2.26)
BMI, kg/m^2^					
(18.5–25)	223 (67%)	58 (75%)	165 (65%)	1	1
< 18.5	33 (10%)	11 (14%)	22 (9%)	1.42 (0.64–3.11)	0.93 (0.35–2.44)
≥ 25	76 (23%)	8 (10%)	68 (27%)	0.33 (0.15–0.74)	0.37 (0.14–0.97)
Albumin, g/dL					
≥ 3.5	254 (77%)	40 (53%)	214 (84%)	1	1
< 3.5	77 (23%)	36 (47%)	41 (16%)	4.70 (2.68–8.23)	5.24 (2.69–10.20)
Hemoglobin, g/dL					
≥ 12 (f) or ≥ 13 (m)	143 (43%)	19 (25%)	124 (49%)	1	1
< 12 (f) or < 13 (m)	187 (57%)	56 (75%)	131 (51%)	2.78 (1.57–4.96)	2.16 (1.07–4.35)
CRP, mg/dL					
≤ 12	232 (75%)	39 (56%)	193 (81%)	1	1
> 12	76 (25%)	31 (44%)	45 (19%)	3.41 (1.92–6.04)	3.25 (1.55–6.81)
Ferritin, ng/mL					
≤ 150 (f) or ≤ 200 (m)	213 (64%)	38 (49%)	175 (69%)	1	1
> 150 (f) or > 200 (m)	119 (36%)	39 (51%)	80 (31%)	2.24 (1.34–3.77)	1.72 (0.92–3.21)
IFN-γ, pg/mL					
Q1–Q3 (5.86–49.65)	219 (66%)	49 (64%)	170 (67%)	1	1
Q4 (> 49.65)	113 (34%)	28 (36%)	85 (33%)	1.14 (0.67–1.95)	0.76 (0.42–1.39)
IL-6, pg/mL					
Q1–Q3 (9.17–49.88)	219 (66%)	50 (65%)	169 (66%)	1	1
Q4 (> 49.88)	113 (34%)	27 (35%)	86 (34%)	1.06 (0.62–1.81)	0.80 (0.45–1.42)
TNF-α, pg/mL					
Q1–Q3 (13.53–27.66)	219 (66%)	41 (53%)	178 (70%)	1	1
Q4 (> 27.66)	113 (34%)	36 (47%)	77 (30%)	2.03 (1.20–3.42)	1.66 (0.94–2.95)
IP-10, pg/mL					
Q1–Q3 (610.78–2922.43)	219 (66%)	38 (49%)	181 (71%)	1	1
Q4 (> 2922.43)	113 (34%)	39 (51%)	74 (29%)	2.51 (1.49–4.23)	1.89 (1.05–3.39)
EndoCab IgM, MMU/mL					
Q1–Q3 (29.5–71.5)	233 (70%)	50 (65%)	183 (72%)	1	1
Q4 (> 71.5)	99 (30%)	27 (35%)	72 (28%)	1.37 (0.80–2.36)	1.19 (0.76–2.13)
LPS, pg/mL					
Undetectable	192 (62%)	45 (63%)	147 (61%)	1	1
Detectable	120 (38%)	26 (37%)	94 (39%)	0.90 (0.52–1.56)	2.39 (1.13–5.06)
sCD14, pg/mL					
Q1–Q3 (487100–2800000)	223 (67%)	45 (58%)	178 (70%)	1	1
Q4 (> 2800000)	109 (33%)	32 (42%)	77 (30%)	1.64 (0.97–2.78)	1.40 (0.79–2.46)
CD4/DR+/38+, % CD4 lymphocytes^2^					
Q1–Q3 (16.3–35.3)	90 (75%)	14 (64%)	76 (78%)	1	1
Q4 (> 35.3)	30 (25%)	8 (36%)	22 (22%)	1.97 (0.73–5.31)	2.60 (0.51–13.21)
CD8/DR+/38+, % CD8 lymphocytes^2^					
Q1–Q3 (36.1–56.9)	90 (75%)	14 (64%)	76 (78%)	1	1
Q4 (> 56.9)	30 (25%)	8 (36%)	22 (22%)	1.52 (0.55–4.18)	1.85 (0.47–7.31)

^1^ Adjusted odds ratio represents the independent risk of the added marker characteristic when adjusting for age, sex, treatment, study site, baseline CD4+ T cell count and baseline HIV viral load. Bold numbers signify p <0.05.

^2^ Only measured where viable peripheral blood mononuclear cells were collected, which excluded all population from Thailand and India.

### Multivariable regression and ROC curve analysis

In multivariable logistic regression models adjusting for age, gender, study site, treatment arm, and baseline CD4 count and log_10_ viral load, CRP and IP-10 remained significantly associated with incident TB (aOR for CRP 3.25, 95% CI: 1.55–6.81; aOR for IP-10 1.89, 95% CI: 1.05–3.39). Detectable LPS was associated with incident TB in the adjusted but not the unadjusted model (aOR 2.39, 95% CI: 1.13–5.06). More established TB risk factors, including hypoalbuminemia (aOR 5.24, 95% CI: 2.69–10.20) and anemia (aOR 2.16, 95% CI: 1.07–4.35), remained associated with incident TB in adjusted models. Being overweight or obese versus normal BMI was inversely associated with incident TB (aOR 0.37, 95% CI: 0.14–0.97).

**Fig 2:**
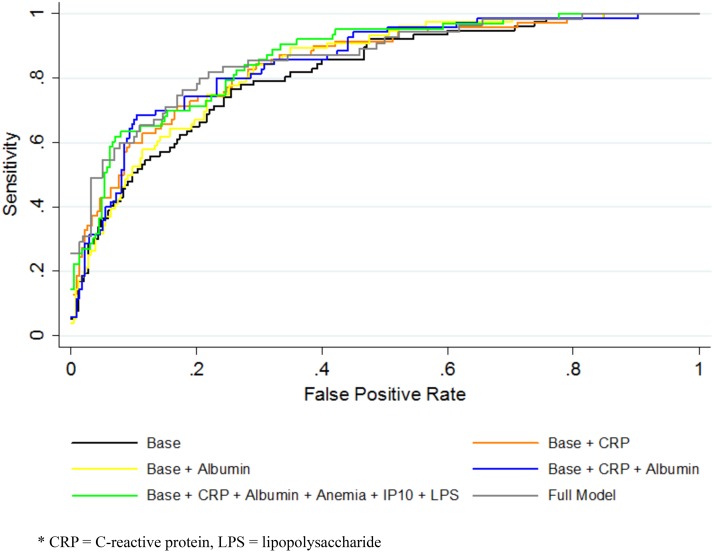
Receiver operating characteristic (ROC) curves.

In ROC analysis, a baseline model including patient characteristics evident from history and physical examination (age, gender, study site, prior history of TB, BMI group) plus pre-ART CD4+ T cell count provided good discrimination of patient risk of post-ART TB (AUC 0.81, 95% CI: 0.76–0.86). (Table 3 and Fig. 2) Hypoalbuminemia, upper quartile CRP, and detectable LPS improved TB risk discrimination the most, albeit minimally, among all biomarkers when added to this model (AUC with hypoalbuminemia 0.84, 95% CI: 0.79–0.89; AUC with CRP 0.85, 95%: CI 0.79–0.90, AUC with detectable LPS 0.84, 95% CI: 0.79–0.89).

**Table 3:** Receiver operating characteristic (ROC) curve analysis for discriminating TB cases from controls.

Model	Cut-off	AUC (95% CI)
Baseline model	*	0.81 (0.76–0.86)
Baseline model plus hypoalbuminemia	Albumin < 3.5 g/dL	0.84 (0.79–0.89)
Baseline model plus anemia	Hemoglobin < 12 g/dL (f) or < 13 g/dL (m)	0.83 (0.78–0.88)
Baseline model plus CRP	CRP > 12 mg/dL	0.85 (0.79–0.90)
Baseline model plus hyperferritinemia	Ferritin > 150 ng/mL (f) or > 200 ng/mL (m)	0.81 (0.76–0.87)
Baseline model plus Log_10_ HIV RNA	Log_10_ HIV RNA > 5	0.81 (0.76–0.87)
Baseline model plus IP-10	IP-10 > 2,922 pg/mL	0.83 (0.76–0.89)
Baseline model plus LPS	LPS detectable	0.84 (0.79–0.89)
Baseline model plus sCD14	sCD14 > 28,000,000 pg/mL	0.83 (0.77–0.88)
Baseline model plus TNF-α	TNF-alpha >27.66 pg/mL	0.83 (0.78–0.89)
Baseline + CRP + hypoalbuminemia	Using above cutoffs	0.86 (0.81–0.90)
Baseline + CRP + IP10	Using above cutoffs	0.85 (0.80–0.91)
Baseline + CRP + LPS	Using above cutoffs	0.86 (0.81–0.90)
Baseline + CRP + hypoalbuminemia + TNF-α	Using above cutoffs	0.86 (0.81–0.91)
Baseline model plus all 9 additional variables	Using above cutoffs	0.86 (0.81–0.92)

* Baseline model includes age (continuous), gender, study site, prior history of TB, BMI group (>18.5, 18.5–24.9, ≥ 25), and CD4+ T cell count group.

## Discussion

This case-control study nested within a multicenter trial of 1571 individuals found that three soluble inflammatory and immune activation markers (CRP, IP-10, and LPS), along with established markers of low albumin and anemia, were independently associated with high risk of incident TB following ART initiation. Those diagnosed with active TB within 96 weeks of ART initiation were five times as likely to have low albumin and two to three times as likely to have highest-quartile levels of CRP and IP-10, detectable plasma LPS, and anemia. Our ROC analysis demonstrated that adding CRP, albumin, or LPS to routinely available measures improves discrimination for incident TB risk within 96 weeks. However, the additive discriminatory value of any of these markers was limited and routinely collected patient measures, including CD4 count, BMI, and prior TB history, provided good discrimination alone (AUC 0.81) in determining who developed active TB.

Individuals at high risk of clinical complications following ART initiation may benefit from closer follow-up and intensified TB case finding; unfortunately, tools for identifying these patients remain limited. Low CD4+ T cell count, underweight, and anemia are known predictors of such outcomes [1,41] but are highly prevalent among people starting ART. Accessible tools that could improve our ability to identify the highest-risk patients are sorely needed. Hypoalbuminemia, present in diverse chronic inflammatory states and a known risk factor for HIV disease progression and mortality as well as post-ART TB in resource-limited settings, had the strongest independent association with TB risk in our multinational population. [3,42] Until now, however, only very limited data have been available for CRP, IP-10, or LPS. One previous study (at a single center in Uganda) found that adults with pre-ART CRP >5 mg/L were at increased risk of ART-associated TB within of the first year of treatment, but only eight TB cases were identified during the follow-up period. [6] Our results extend this finding to a much larger and more diverse patient population; of note, the upper quartile of CRP in our population (i.e., the definition of a “positive test”) was substantially higher, at approximately 12 mg/dL.

Importantly, CRP levels at the time of ART initiation may also be associated with non-TB morbidity and mortality. For example, in a study of ART-naïve patients from multiple sites within the United States, individuals with pre-ART CRP greater than the median within the study population were more likely to experience AIDS events and death from all causes within the first year of treatment. [29] Such evidence, taken as a whole with our results here, highlight the importance of recognizing people at greatest risk for both TB and non-TB adverse events soon after ART initiation and suggest that CRP may play a role in identifying high-risk patients even in settings where CD4+ T cell count and other clinical variables are known.

IP-10 was the single pro-inflammatory cytokine or chemokine marker associated with TB risk in our study. IP-10 is a chemokine secreted by multiple cell types, including monocytes, endothelial cells, and fibroblasts, in response to IFN- γ. It acts as a chemoattractant for monocytes/macrophages, T cells, NK cells, and dendritic cells and promotes T cell adhesion to endothelial cells, antitumor activity, and inhibition of bone marrow colony formation and angiogenesis. [43,44] IP-10 concentrations, measured after *M*. *tuberculosis* antigen exposure or in unstimulated blood, are elevated in patients with TB either with or without HIV co-infection. [13–23] Our study is the first to our knowledge to associate IP-10 concentration with subsequent risk of TB disease in populations starting ART. IP-10 was recently identified as a marker of rapid disease progression in newly diagnosed HIV infection and was associated with severity of hepatitis C in those with HIV co-infection. [45,46] Like CRP, IP-10 may therefore be a risk factor not only for TB but also for other non-TB adverse outcomes. The underlying mechanism linking CRP and IP-10 levels to reactivation of TB following initiation of ART however remains uncertain. Their elevation could represent a prodromal immunologic response to nascent infection or an inflammatory milieu characterized by CRP and IP-10 elevations that is ripe for reactivation of TB. Future time studies will need to characterize associated immunologic process in more detail in order to address these possible scenarios.

The association between bacterial translocation and host risk of incident TB post-ART has not been characterized. HIV infection is associated with reduced intestinal mucosal integrity and microbial translocation, the magnitude of which predicts HIV disease progression and response to ART. [26] LPS, a cell wall component of gram-negative bacteria, is a principle biomarker for bacterial translocation. Disruption of the gastrointestinal mucosal barrier leads to translocation of microbial products including LPS, triggering downstream immune activation. We found that detectable levels of LPS in patients was associated with a 2-fold risk of incident TB post-ART. Plausibly, circulating LPS might lead to greater TB risk through a number of means, including by impeding normal immune reconstitution after ART initiation.

Our analysis has certain limitations. First, as several of the novel soluble markers studied have no established cutoff values when used for predicting incident TB, and in this population of HIV-infected adults, we used the upper quartile of most markers to provide fair comparison and employed single-site quality-assured laboratories to maximize interpretability. Second, our study involved trial participants who are likely to be healthier and more fastidious in taking ART than HIV-infected populations in programmatic settings. [47] Third, as a retrospective analysis, we were unable to prospectively evaluate the use of these biomarkers in changing actual clinical practice. Fourth, interferon-gamma release assays (IGRAs) or tuberculin skin tests (TSTs) were not routinely performed in our study population. Further evaluation of biomarkers by positive or negative IGRA/TST result could further stratify TB risk and provide insight into whether markers are more likely a measure of TB susceptibility or rather predictive of TB reactivation after ART. However, the study was done primarily in high-burden settings where latent TB is already very common and is not a highly predictive marker of progression to active TB. [48]

However, our study is among the first to evaluate the clinical utility of novel soluble biomarkers in predicting incident TB among a diverse but well characterized and well monitored group of HIV-infected adults residing in low- and middle-income countries.

Taken as a whole, these findings suggest that CRP, IP-10, and LPS, and albumin could play a role in identifying patients at high risk of HIV-related complications including TB early after ART initiation. Notably, these markers are not pathogen-specific for *Mycobacterium tuberculosis* but may instead signal high susceptibility for multiple adverse events after ART initiation and provide better discrimination than we observed in determining pooled TB and non-TB outcomes. Such patients may be candidates for more intensified follow-up after starting ART, especially as follow-up algorithms move away from a “one size fits all” paradigm and toward a more individualized approach. Relative to HIV RNA levels and even CD4 counts, several of these markers may be simpler to measure. CRP is available as a low-cost point of care test, IP-10 can be measured using commercially available assays, and serum albumin testing is already widely available. Our results thus suggest the need for further studies to investigate the most appropriate cutoffs of these biomarkers, evaluate their associations with early adverse events other than TB, and study the cost-effectiveness of specific clinical algorithms that use these tests to individually tailor therapy and follow-up after ART initiation.
